# Immune-related aseptic meningitis and strategies to manage immune checkpoint inhibitor therapy: a systematic review

**DOI:** 10.1007/s11060-022-03997-7

**Published:** 2022-04-13

**Authors:** Simon Nannini, Larysa Koshenkova, Seyyid Baloglu, Dominique Chaussemy, Georges Noël, Roland Schott

**Affiliations:** 1 Department of Medical Oncology, Strasbourg-Europe Cancer Institute (ICANS), 67200 Strasbourg, France; 2 Department of Radiology, Strasbourg University Hospital 67033, Strasbourg, France; 3 Department of Neurosurgery, Strasbourg University Hospital 67033, Strasbourg, France; 4 Department of Radiation Oncology, Strasbourg-Europe Cancer Institute (ICANS), 67200 Strasbourg, France

**Keywords:** Immune-related adverse event, Immunotherapy, Reintroduction, Aseptic meningitis, Melanoma

## Abstract

**Introduction:**

Immune checkpoint inhibitors (ICIs) can induce adverse neurological effects. Due to its rarity as an adverse effect, meningitis has been poorly described. Therefore, meningitis diagnosis and management can be challenging for specialists. Moreover, meningitis can be an obstacle to resuming immunotherapy. Given the lack of alternatives, the possibility of reintroducing immunotherapy should be discussed on an individual basis. Here, we present a comprehensive systematic review of meningitis related to ICIs.

**Review:**

We performed a search for articles regarding immune-related meningitis published in PubMed up to November 2021 with the MeSH terms “meningitis” and “immune checkpoint” using the Preferred Reporting Items for Systematic Reviews and Meta-Analyses (PRISMA) method. We summarized the studies not only by category but also based on whether it was a primary article or case report to provide a systematic overview of the subject. We reviewed a total of 38 studies and herein report the clinical experiences, pharmacovigilance data and group knowledge from these studies.

**Conclusion:**

This review summarizes the existing information on immune-related meningitis and the possibility of reintroducing immunotherapy after the development of central neurological side effects. To the best of our knowledge, there is little information in the literature to guide clinicians on decisions regarding whether immunotherapy should be continued after a neurological adverse event occurs, especially meningeal events. This review emphasizes the necessity of systematic examinations, steroid treatment (as a cornerstone of management) and the need for further exploratory studies to obtain a clearer understanding of how to better manage patients who experience these side effects. The findings summarized in this review can help provide guidance to practitioners who face this clinical situation.

## Introduction

Currently, immune checkpoint inhibitors (ICIs) have become the standard of care for numerous cancers. In 2011, ipilimumab was approved by the Food and Drug Administration (FDA) to treat metastatic melanoma (MM), with an improvement in progression-free survival (PFS) of 4 months [1]. In 2015, nivolumab, an inhibitor of programmed death ligand 1 (PDL1), improved the overall response of MM patients compared to dacarbazine [2]. In 2017, the combination of nivolumab and ipilimumab achieved a median overall survival (OS) of 60 months compared to the 36.9 months achieved with nivolumab alone for the treatment of MM [3]. Consequently, the nivolumab plus ipilimumab combination became the new standard of care for BRAF-negative MM.

However, ICIs induce unique side effects. Ipilimumab alone and its combination with nivolumab are associated with the highest rates of immune-related adverse effects (irAEs) among other immunotherapies, as 53% of patients treated with such regimens had grade 3–4 irAEs [4]. IrAEs can involve the central nervous system (CNS) and are often severe despite their rarity. Due to the difficulty in diagnosing neurological irAEs, the reported incidence of 1–5% is probably an underestimate [5]. In particular, immune-induced aseptic meningitis is associated with high rates of mortality and/or morbidity [7]. Systematic explorations with at least CNS imaging, lumbar puncture, viral screening and viral serology analysis are recommended by the European Society for Medical Oncology (ESMO) [8]. If meningeal irAEs cause sufficient concern, management typically features high-dose steroid administration for at least 4 to 6 weeks with decreasing doses [8].

Whether ICIs should be resumed thereafter is still debated. After some irAEs develop, because of the lack of an efficient alternative option for metastatic disease treatment, resuming ICIs can be the best choice. The current review attempted to summarize reported knowledge about the management of immune-related meningitis and the reintroduction of ICIs.

## Methodology

**Fig.1:**
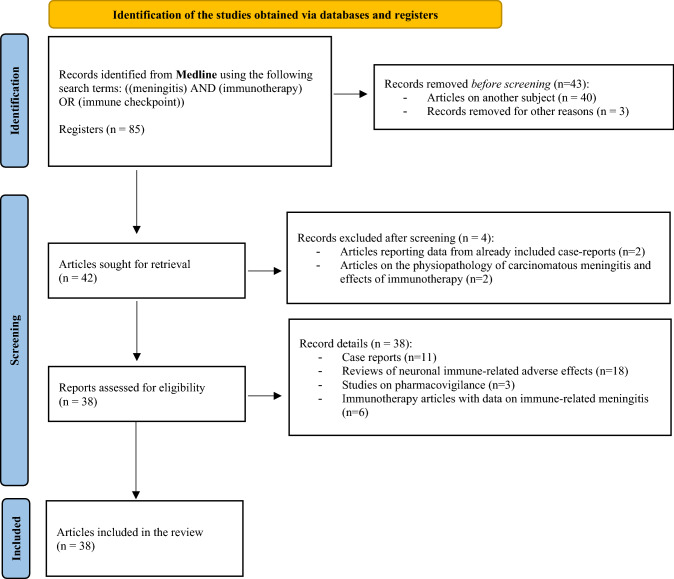
PRISMA flow diagram of the literature search strategy

We searched for articles related to immune-related meningitis published on PubMed with the MesH terms “meningitis” and “immune checkpoint” up to November 19, 2021, using the Preferred Reporting Items for Systematic Reviews and Meta-Analyses (PRISMA) method (Fig. 1.). We summarized primary articles and case reports to give a systematic overview of the subject.

## Results

**Fig. 2:**
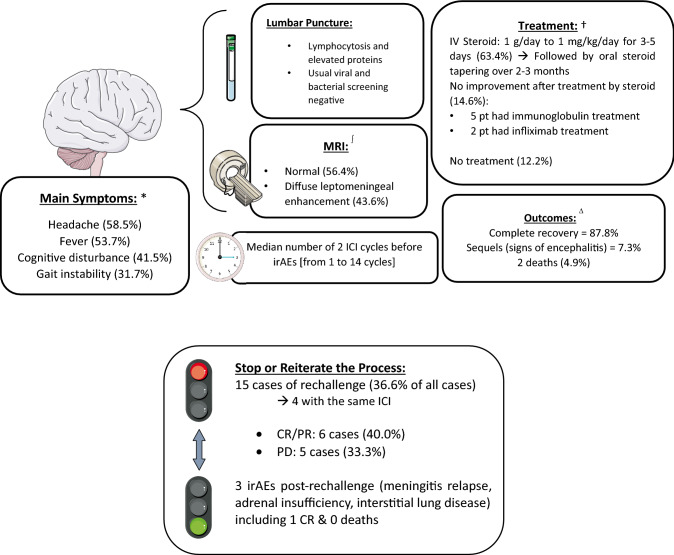
Summary of the 41 cases reported in this review. * = percentages of the symptoms reported in the 41 cases; patients could have more than one symptom; ∫ = percentages from the case reports including MRI results (n = 39); † = percentages from the case reports on treatment initiation (n = 41); ∆ = percentages from the case reports about the patient course after diagnosis of irAEs (n = 41). Abbreviations: *CR* complete response, *PD* progressive disease, *PR* partial response, *p*t patients, *ICI* immune checkpoint inhibitor, *irAEs*  immune-related adverse events, *IV*  intravenous, *MRI* magnetic resonance imaging. The figure was generated with illustrations from smart.servier.com

In 11 articles, 40 cases of immune-related meningitis or meningoencephalitis (with at least signs of meningitis on lumbar puncture) were reported [10–22] (Tables 1 and 2). An overview of the results is presented in Fig. 2. In our systematic review, 18 articles were reviews of neuronal irAEs. Their main points are summarized in the following sections in parallel with a description of the case series.

**Table 1:** Case reports on immune-related meningitis: patient characteristics and clinical and paraclinical signs

References	Sex	Age (years)	Tumor type	ICI received	Time to 1st signs of meningitis	Symptoms	Lumbar puncture results	MRI results
Cuzzubbo S et al. [11]	M	71	Stage IIIc melanoma	Nivo 3	6 days after the 1st cycle	Fever, partial seizure and confusion	Cytology: 40 cells/mm3 with 90% lymphocytes—protein content = 0.99 g/L	Nonspecific
	F	29	Stage IIIc melanoma	Ipi 1—Nivo 3	6 days after the 1st cycle	Headache, nausea and photophobia	Cytology: 8 cells/mm3 with 100% lymphocytes—protein content = 0.30 g/L	Nonspecific
	F	51	Stage IV melanoma	Spartalizumab 400 mg	95 days after the 1st cycle	Headache and pain in 4 limbs	Cytology: 19 cells/mm3 with 90% lymphocytes—protein content = 0.39 g/L	Nonspecific
	F	46	Stage IV melanoma	Ipi 1—Nivo 3	50 days after the 1st cycle	Headache and vomiting	Cytology: 25 cells/mm3 with 90% lymphocytes—protein content = 0.43 g/L	Nonspecific
	F	64	Stage IIc melanoma	Nivo 3	6 days after the 1st cycle	Headache and vomiting	Cytology: 0 cells/mm3—protein content = 0.59 g/L	Nonspecific
	M	27	Stage IIIc melanoma	Ipi 3 – Nivo 1	9 days after the 1st cycle	Headache and fever	Cytology: 9 cells/mm3 with 90% lymphocytes—protein content = 0.54 g/L	Nonspecific
	F	20	Stage IV melanoma	Ipi 3—Nivo 1	17 days after the 1st cycle	Headache and fever	Cytology: 320 cells/mm3 with 90% lymphocytes—protein content < 0.45 g/L	Nonspecific
Thouvenin L et al. [19]	F	46	Stage IV uveal melanoma	Ipi 3	4 cycles after the reintroduction of ICI after the development of hypophysitis	Headache, hearing loss, nausea, asthenia, slightly elevated temperature, and cerebellar syndrome	Cytology: elevated cells/mm3 with 91% lymphocytes—elevated protein content	Regressive sequelae of hypophysitis
	M	70	Stage IV renal cell carcinoma	Ipi 3—Nivo 1	5 days after the 1st cycle	Neck pain, fever, gait disturbance, aphasia and confusion	Cytology: elevated cells/mm3 with 66% lymphocytes—elevated protein content	Ventriculitis
	F	44	Stage IV MSI colorectal carcinoma	Ipi 1—Nivo 3	After 3 cycles	Headache, fever, and photophobia	Cytology: elevated cells/mm3 with 92% lymphocytes—elevated protein content	Nonspecific
	M	82	Recurrent Hodgkin's lymphoma	Pembrolizumab 200 mg	10 days after the 1st cycle	Confusion, impaired speech, gait disturbance, and fever	Cytology: elevated cells/mm3 with 91% lymphocytes—elevated protein content	Multiple areas with contrast and leptomeningeal enhancement
	M	68	Stage IV renal cell carcinoma	Ipi 1—Nivo 3	After 3 cycles of Ipi-Nivo and 1 cycle of Nivo alone	Fever, speech disturbance, confusion, and drowsiness	Cytology: elevated cells/mm3 with 99% lymphocytes—elevated protein content	Diffuse dural enhancements
	F	19	Stage IV melanoma	Ipi 1—Nivo 3	After 3 cycles	UNK	Cytology: elevated cells/mm3 with 97% lymphocytes—elevated protein content	Nonspecific
	F	70	Stage IV renal cell carcinoma	Ipi 1—Nivo 3	After 2 cycles	Headache, nausea, and dizziness	Cytology: elevated cells/mm3 with 99% lymphocytes—elevated protein content	Nonspecific
	M	56	Stage IV uveal melanoma	Ipi 3	After 4 cycles	Nausea, asthenia, fever, gait imbalance, hallucinations, and myoclonic jerking	Cytology: elevated cells/mm3 with 96% lymphocytes—elevated protein content	Diffuse dural enhancements
	M	55	Stage IV lung adenocarcinoma	Pembrolizumab 200 mg	After 11 cycles	Headache and photophobia	Cytology: elevated cells/mm3 with 30% lymphocytes—elevated protein content—high opening pressure	Nonspecific
	F	53	Stage IV melanoma	Ipi 3—Nivo 1	After 2 cycles	Fever, aphasia, dizziness, asthenia, and slurred speech	Cytology: elevated cells/mm3 with 86% lymphocytes—elevated protein content	Nonspecific
	M	61	Stage IV melanoma	Ipi 3 – Nivo 1	After 4 cycles of Ipi-Nivo and 1 cycle of Nivo alone	Altered mental status	Cytology: elevated cells/mm3—elevated protein content	Nonspecific
	M	57	Stage IV melanoma	Nivo 3 follow by Ipi 3	After 14 cycles of Nivo alone and 4 of Ipi alone	Headache and confusion	Cytology: elevated cells/mm3 (lymphocytosis)—elevated protein content	Nonspecific
	UNK	UNK	Stage IV melanoma	Ipi	After 2 cycles	Headache, nausea, vomiting, and drowsiness	Cytology: few lymphocytes	UNK
	UNK	UNK	Stage IV melanoma	Ipi—Nivo	After 2 cycles	Headache and nausea	Cytology: reactive lymphocytes	UNK
	F	71	Stage IV lung adenocarcinoma	Pembrolizumab	After 6 cycles	Diplopia, gait disturbance, and lower limb paresthesia	Cytology: elevated cells/mm3 (lymphocytosis)—elevated protein content—positive anti-Rib antibody	Nonspecific
	M	20	Recurrent Hodgkin's lymphoma	Nivo 3	After 3 cycles	Headaches, diplopia, confusion, nausea, vomiting, ataxia, and dysmetria	Cytology: elevated cells/mm3 with 94% lymphocytes—elevated protein content	Cerebellar edema
	M	63	Stage IV renal cell carcinoma	Nivo 300 mg	After 6 cycles	Uncontrolled choreatic movements	Cytology: mild inflammation—positive anti-PNMA2 antibody—autopsy focal lymphocytic meningitis of the entire brain and cervical spinal cord	Pathological increased signal within the basal ganglia
	M	51	Stage IV squamous lung carcinoma	Pembrolizumab	After 8 months	Fever, headache, ataxia, and Kernig sign	Cytology: elevated cells/mm3 (lymphocytosis)—elevated protein content	Nonspecific
	M	56	Stage III melanoma	Adjuvant Ipi 10	After 4 cycles	Dizziness, neck pain, headache, and severe gait ataxia	Cytology: elevated cells/mm3 with 99% lymphocytes—elevated protein content	Arachnoiditis
	F	39	Stage IIIA melanoma	Adjuvant Ipi 10	After 3 cycles	Headache and flu-like symptoms	Cytology: elevated cells/mm3 (lymphocytosis)—elevated protein content—high opening pressure	Leptomeningeal enhancement and pituitary enlargement
	M	51	Stage IV melanoma	Ipi 3	After the 1st cycle	Headache and fever	Cytology: elevated cells/mm3—elevated protein content—high opening pressure	Nonspecific
	F	45	Stage IV melanoma	Ipi 3	After 3 cycles	Confusion, headache, nausea, and dysmetria	Cytology: elevated cells/mm3—elevated protein content—high opening pressure	Nonspecific
Toyozawa R et al.—JTO Clin Res Rep. 2020 [22]	F	71	Stage IV lung carcinoma	Atezolizumab (+ carboplatin + paclitaxel + bevacizumab)	14 days after the 1st cycle	Fever and disturbance of consciousness	Cytology: normal cells/mm3—protein content = 1.36 g/L	Nonspecific
	M	55	Stage IV lung adenocarcinoma	Atezolizumab	11 days after the 1st cycle	Fever and disturbance of consciousness	Cytology: normal cells/mm3—protein content = 1.30 g/L	Nonspecific
	M	50	Stage IV lung adenocarcinoma	Atezolizumab	11 days after the 1st cycle	Fever and disturbance of consciousness	Cytology: 15 cells/mm3—protein content = 3.58 g/L	Abnormal enhancements along the lines of the corpus callosum
Ogawa K et al. [18]	M	56	Stage IV lung adenocarcinoma	Atezolizumab after 14 cycles of Nivo	11 days after the 1st cycle	Fever, headache, asthenia, and dysarthria	Cytology: 25 cells/mm3—protein content = 1.34 g/L	Meningeal enhancement
Minami S et al. [17]	F	65	Stage IV lung adenocarcinoma	Pembrolizumab	After 13 cycles (8 months)	Asthenia, chills, and fever	Cytology: 197 cells/mm3 (97% mononuclear cells)—protein content = 0.32 g/L	Nonspecific
Shields LBE et al. [16]	M	66	Stage IV renal cell carcinoma	Nivo 240 mg	After 7 cycles	Bilateral lower extremity weakness, lethargy, fever, confusion, and coma	Cytology: 27 cells/mm3 (78% mononuclear cells)—elevated protein content	Diffuse leptomeningeal enhancements
Yonenobu Y et al. [15]	M	61	Stage IV squamous lung carcinoma	Pembrolizumab	After 2 cycles	Consciousness disturbance	Cytology: 79 lymphocytes/mm3—protein content = 2.09 g/L	High signal intensity lesions in the left frontal lobe and pons
Laserna A et al. [14]	F	53	Stage IV squamous lung carcinoma	Atezolizumab	13 days after the 1st cycle	Altered mental status, headache, meningeal signs and coma	Cytology: 553 mcL (91% PNNs)—protein content > 6 g/L	Diffuse leptomeningeal enhancements
Bello-Chavolla OY et al. [13]	M	66	Stage IV melanoma	Ipi 10 follow by Ipi 10—Nivo 3	3 days after the last cycle; after 9 cycles of Ipi alone and 4 cycles of Ipi-Nivo	Fever, generalized weakness, headache, and hyporexia	No lumbar puncture (patient refusal)	Not performed
Ohno N et al. [12]	M	76	Stage IV renal cell carcinoma	Ipi 1—Nivo 3	After 2 cycles	Consciousness disturbance, and fever	Cytology: 147 cells/mm3—protein content = 3.85 g/L	Diffuse meningeal enhancement
Katakura Y et al. [21]	M	58	Stage IV melanoma	Nivo followed by Ipi—Nivo	After 3 cycles of Nivo alone and 1 cycle of Ipi-Nivo	Fever and headache	Mononucleosis-significant cell number increase—No data about protein content	Not performed

*F* female, *Ipi* ipilimumab, *Ipi* 1 1 mg/kg ipilimumab, *Ipi* 10 10 mg/kg ipilimumab, *M* male, *Nivo* nivolumab, *Nivo* 3 3 mg/kg nivolumab, *UNK* unknown

**Table 2:** Case reports about immune-related meningitis: patient treatment and follow-up

References	Treatment of irAEs	Response	Treatment reintroduction	Reintroduced treatment	Best response after irAEs	Patient course
Cuzzubbo S et al. [11]	Steroids 1 mg/kg/day for 7 days followed by 42 days of tapering	Complete recovery 2 days after steroid treatment and 18 days after the 1st signs	Yes—373 days after initial treatment	Ipi 1—Nivo 3 (0,5 mg/kg/J steroids)	PD	PD at 3 months and death from cancer progression
	Steroids 1 mg/kg/day for 7 days followed by 42 days of tapering	Complete recovery 14 days after steroid treatment and 17 days after the 1st signs	Yes—54 days after initial treatment	Ipi 1—Nivo 3	CR	CR at 32 months after reintroduction
	No treatment	Complete recovery in 10 days	Yes—24 days after initial treatment	Spartalizumab	PD	Grade 3 interstitial lung disease and PD 3 months after reintroduction
	Steroids 1 mg/kg/day for 7 days followed by 42 days of tapering	Complete recovery 2 days after steroid treatment and 21 days from the 1st signs	Yes—118 days after initial treatment	Nivo 3	PD	PD at 3 months and death from cancer progression
	No treatment	Complete recovery in 65 days	Yes—4 days after initial treatment	Nivo 3	PR	PR at 3 months, maintained at 6 months
	Steroids 1 mg/kg/days for 14 days followed by 42 days of tapering	Complete recovery 14 days after steroid treatment and 49 days from the 1st signs	Yes—126 days after initial treatment	Spartalizumab + ribociclib	PD	PD at 3 months and death from cancer progression
	No treatment	Complete recovery in 10 days	Yes—19 days after initial treatment	Nivo 3	PR	PR at 3 months, maintained at 17 months
Thouvenin L et al. [19]	IV steroids 4 mg/kg/J for 6 days followed by 6 weeks of oral steroid tapering	Improvement and relapse 1 week after steroid treatment > improvement and remission after treatment with 12 mg/day oral dexamethasone > tapering over 3 months	Yes—only after 2 years and disease progression	Pembrolizumab 2 mg/kg	PR	PR for 2 years—pembrolizumab given at disease progression without irAE—death 8 months after treatment with new ICI
	IV steroids 1,8 mg/kg/J for 7 days followed by 6 weeks of oral steroid tapering	Improvement in a few days but long tapering because of several recurrences (total of 7 months)	No	No	PR	PR for 7 months and pazopanib administered after relapse
	IV steroids 2 mg/kg/J for 3 days followed by 6 weeks oral steroid tapering	Complete recovery after 3 days of steroid treatment	Yes—shortly resumed after steroid discontinuation	Nivo 3	PR	Dissociated radiological response, no IrAE recurrence
	IV steroids 1 mg/kg/J for 5 days followed by 3 months of oral steroid tapering	Complete recovery in a few days after steroid treatment	No	No	CR	CR without new treatment
	Oral steroids for 7 days followed by 1 month of tapering	Complete recovery	No	No	SD	SD at 9 months
	IV steroids for 8 days followed by 1 month of oral steroid tapering	Complete recovery	Yes—3 months after resolution	UNK	PD	PD
	IV steroids 1 mg/kg/J and 1 month of oral steroid tapering	Complete recovery	Yes—3rd cycle at 10 mg/J steroids	Ipi 1—Nivo 3	CR	Adrenal insufficiency, recurrence of meningitis and hepatitis after the 3rd cycle—no ICIs were administered, but CR was achieved
	IV steroids followed by 4 months tapering	Improvement in 48 h	No	No	UNK	UNK
	IV steroids followed by oral steroid tapering	Complete recovery in 1 day	No	No	CR	CR
	IV steroids, but no tapering data	Complete recovery after 3 days of steroid treatment	Yes—after PD during treatment with dabrafenib-trametinib	Pembrolizumab	PD	PD without irAEs
	IV steroids for a few days; the second treatment was combined with IG followed by oral steroid tapering	Complete recovery only after increased steroid and IG dose	No	No	PD	PD at 4 months
	IV steroids followed by oral steroid tapering	Complete recovery in 6 days	No	No	PR	VGPR
	No treatment	Complete recovery in 10 days	UNK	UNK	PD	PD at 6 months
	No treatment	Complete recovery in 7 days	UNK	UNK	PR	PR for 16 months
	Oral steroids for 12 weeks	Complete recovery at 8 weeks > relapse 3 weeks after steroid treatment; treated with rituximab and IV steroids > relapse under steroid treatment after 4 months; addition of cyclophosphamide	No	No	CR	CR
	Steroids for 4 weeks	Recovery at days 6 except for diplopia	No	No	PR	PR
	IV steroids with addition of infliximab at deterioration	Cognitive deterioration	No	No	UNK	Death due to irAE
	IV steroids with 10% tapering per week	Improvement in a few days except for ataxia	No	No	SD	SD at 1 year
	IV steroids for 3 days follow by IG for 5 days after the development of worsening neurological symptoms (ultimately resulting in tetraplegia), subsequent administration of oral steroids for 4 months	With IG and IV steroids, improvement over 1 month, but complete recovery only after 24 months	No	No	UNK	UNK
	IV steroids and oral steroid tapering over 8 weeks; relapse treated with IV steroids, IG and infliximab with steroid tapering over 3 months	Rapid improvement of the first signs of disease; near complete recovery of relapse only after infliximab treatment	No	No	UNK	UNK
	Oral steroids	Complete recovery in a few days after steroid treatment	UNK	UNK	SD	SD at 10 months
	Oral steroids, IV steroids after deterioration, and then IG	Improvement only after IG treatment	UNK	UNK	UNK	UNK
Toyozawa R et al.—JTO Clin Res Rep. 2020 [22]	IV steroids, but no tapering data	Complete recovery	UNK	UNK	UNK	UNK
	IV steroids, but no tapering data	Improvement after 2 days	UNK	UNK	UNK	UNK
	IV steroids, but no tapering data	Complete recovery	UNK	UNK	UNK	UNK
Ogawa K et al. [18]	IV steroids 1 g/day for 3 days and 12 weeks of oral steroid tapering	Improvement after 3 days	No	No	SD	SD at 3 months
Minami S et al. [17]	IV steroids 1 g/body/day	Death after 5 days	No	No	UNK	Death after 5 days
Shields LBE et al. [16]	Oral steroids 90 mg for 6 days follow by tapering	Complete recovery after 2 weeks	No	No	SD	SD after 40 months
Yonenobu Y et al. [15]	IV steroids 1 g twice for 3 days follow by oral steroid 1 mg/kg and IG	Improvement in a few days	UNK	UNK	UNK	UNK
Laserna A et al. [14]	IV steroids 15 days and tapering over 19 days	Improvement after 15 days of IV steroid treatment	UNK	UNK	UNK	UNK
Bello-Chavolla OY et al. [13]	IV steroids 1 g/day for 3 days followed by tapering	Complete recovery after 2 days	Yes	Nivo 3	UNK	UNK
Ohno N et al. [12]	IV steroids and oral steroid tapering	Improvement within a few days of IV steroid treatment, but the polyradiculo-neuropathy remained with antiganglioside antibodies	UNK	UNK	UNK	UNK
Katakura Y et al. [21]	30 mg prednisolone and gradual tapering over 6 months	Complete recovery	Yes	Nivo	PR	Adrenal insufficiency, PR at 55 weeks after rechallenge

*CR* complete response, *IG* intravenous immunoglobulin, *irAE* immune-related adverse event, *IV* intravenous, *PD* progressive disease, *PR* partial response, *SD* stable disease, *UNK* unknown

### Population characteristics

Data from 40 patients, including 22 men and 16 women with a median age of 56 years, were collected [range 19–82 years]. Overall, 21 patients (52.5%), 10 patients (25.0%), six patients (15.0%), two patients and one patient presented with melanoma, lung cancer, renal cell carcinoma, Hodgkin lymphoma, and colorectal cancer with microsatellite instability, respectively. Four patients (10.0%) had brain metastasis, and surgery was performed on one patient, but no other data on local treatment were reported for the other patients.

Ipilimumab and nivolumab were the most frequently prescribed ICIs. The combination of both was used in 16 patients (40.0%), ipilimumab alone was used in seven patients (17.5%), and nivolumab alone was used in five patients (12.5%). Pembrolizumab was used in six patients (15.0%), atezolizumab was used in five patients, and spartalizumab was used in one patient.

### Clinical outcomes

The most common symptoms were headache, fever, cognitive disturbance and gait instability. The symptoms began after a median of 2 cycles [range 1–14 cycles]. The clinical status of patients deteriorated quickly, occurring within a few days after the beginning of symptoms. All patients except three presented with cerebrospinal fluid (CSF) lymphocytosis. One patient refused lumbar puncture, and one did not have detectable cells in the CSF [11], and their last exam showed only a protein content over 6 g/L [14]. Data on the white blood cell count was available for 17 patients, with a median value of 25 cells/mm^3^ (0–320 cells/mm^3^). Proteinorachy was described for 16 patients, with a median value of 0.87 g/L (0.3–3.85 g/L). Cerebral imaging was performed by magnetic resonance imaging (MRI) for 38 patients, with diffuse leptomeningeal enhancement observed in 16 (42.1%). One patient had cerebral edema, which is a sign of encephalitis [19]. No specific signs were reported for 21 patients (55%). Some cases reported specific contrast enhancement of the basal ganglia, pituitary gland, corpus callosum or frontal lobe.

### Treatment and evaluation

Proper tapering of high-dose corticosteroids is the cornerstone of treatment [70]. Unfortunately, 20% of patients did not improve with corticosteroids alone, and the addition of an immunosuppressive agent was required [71, 72]. Due to the potential residual benefit of ICIs, multidisciplinary discussions and decisions, particularly about the management of severe cases, are important, especially when the patient is in intensive care [73].

In our case series, the main treatment component was steroids. 30 patients received intravenous (IV) steroids (75.0%), and five received oral steroids (12.5%). The initial dose varied between 1 g/day and 1 mg/kg/day for 3 to 5 days, followed by a dose reduction over a median of 6 weeks after improvement. Katakura et al. reported a patient treated with 30 mg of oral steroids but did not specify the time to complete recovery.

In six patients (15.0%), symptoms did not improve after steroid treatment. IV immunoglobulins were administered to five patients, and infliximab was introduced to two patients. Garcia et al. reported a patient who improved after IV steroid administration but quickly relapsed at the end of the steroid decrease. Consequently, a combination of steroids and immunoglobulins was tried, but the outcome was unsatisfactory. The addition of infliximab finally induced a near complete recovery [23]. Thouvenin et al. reported the case of a 63-year-old man treated with nivolumab for renal cell carcinoma who developed immune-related meningoencephalitis with uncontrolled choreatic movements. Despite steroid and infliximab treatments, the patient deteriorated and died [19].

After the initiation of the treatment, improvement usually occurred in a few days. However, Bompaire et al. reported a case of severe meningoneuritis that required IV steroids and immunoglobulin, which induced symptom improvement within only 1 month. The patient remained in complete remission after 24 months [24]. Sequelae-free complete recovery was observed in 35 patients (87.5%). Only three patients (7.5%) did not achieve complete symptom improvement. All of these patients had clinical signs more related to encephalitis (ataxia and diplopia) [25, 26] or polyradiculoneuropathy [12] than to meningitis. Kopecky et al. and Minami et al. reported two cases of death due to meningitis (4.9%). Both patients died quickly, 1 week after the beginning of deterioration, despite the start of high-dose steroids and/or infliximab [17, 27].

In five cases, the authors did not administer treatment because of low-grade meningitis. Spontaneous improvement was noted at a median time of 10 days (7–65 days) [11, 19].

### Follow-up and therapy reintroduction

After recovery, ICI reintroduction was proposed in 14 patients (35.0%). In four patients, the same ICI was prescribed. New irAEs were reported in three patients after reintroduction, all of whom had received the same ICI. One patient developed interstitial lung disease and meningitis relapse, and the other two developed adrenal insufficiency [11, 21, 28]. Takamasu et al. reported that a patient with stage IV renal cell carcinoma achieved a complete response owing to the combination of ipilimumab 1 mg/kg and nivolumab 3 mg/kg, despite irAE reoccurrence [28]. Six of the seven cases reported by Cuzzubbo et al. did not experience irAE reoccurrence, even after ICI continuation, with two of the six cases receiving dual ICI treatment with 1 mg/kg ipilimumab. The patient treated with spartalizumab was diagnosed with interstitial lung disease shortly after reintroduction of the same ICI [11]. Fellner et al. also reported successful outcomes after the reintroduction of ICIs, but only with nivolumab, as irAEs developed with the combination of ipilimumab and nivolumab [81].

Five patients who received therapy reintroduction (35.7%) demonstrated progressive disease, and three of these patients succumbed to disease-related death. Five patients (35.7%) had a complete or partial response, and one other had a dissociated response. No stable disease was reported in the therapy reintroduction population.

At the last follow-up after irAEs were reported, among the patients with reported data, the overall response rate was 51.9%. Five patients achieved a complete response (18.5%), and nine patients achieved a partial response (33.3%). Eight patients experienced disease progression (29.6%), and five patients had stable disease (18.5%). The disease control rate was 70.4%, which is comparable to the rates reported in phase 3 studies of immunotherapy [3, 29].

### Pharmacovigilance studies

Three articles analyzed pharmacovigilance data using disproportionality analysis, and the results revealed an association between ICI use and neurotoxicity [7, 30, 31]. Johnson et al. reported 18,518,994 neurological AEs, among which 48,653 were related to ICIs. The researchers concluded that the patients receiving ICIs had a higher incidence of myasthenia gravis (ROR = 16.5), encephalitis (ROR = 10.4), peripheral neuropathy and meningitis compared to those receiving other systemic treatments (ROR = 3.1). Meningitis (0.15% of patients in their cohort) was preferentially associated with the use of anti-CTLA-4 agents [7].

Sato et al. reported data from the Japanese Adverse Drug Event Report database. From a total of 7604 cases of irAEs, they identified 583 (7.67%) neurological AEs related to ICIs. The authors compared the incidences of AEs between nivolumab and other ICI subtypes. They concluded that the use of ipilimumab was associated with a higher incidence of meningitis. The time to the development of meningitis was shorter than the time to the development of other neurological irAEs [31]. In another study of 50,406 irAEs by Mikami et al., they used the FDA reporting system and identified 3619 neurological irAEs (7.2%). This number is similar to that reported by Sato et al., but Mikami et al. showed a higher incidence of neurological complications with the use of ICIs than non-ICI drugs. ICI combinations were associated with a higher incidence of neurological complications, mainly hypophysitis and hypopituitarism. The authors do not report any other risk factors associated with this higher incidence. Dual ICI therapy, older age, melanoma and non-small-cell lung cancer (NSCLC) seemed to be associated with a higher risk of fatal neurological irAEs, including meningeal irAEs [30].

### ICI efficacy in brain and leptomeningeal metastasis

Of the studies retrieved by our literature search, five articles focused on the efficacy of ICIs in patients with central nervous system metastasis. Kuske et al. reviewed different treatments for melanoma brain metastasis and reported on phase 2 studies that evaluated ICIs in brain metastasis, which showed an intracranial response of approximately 42 to 55%. No difference in safety data was reported, except for slightly more headaches of any grade with dual ICI treatment [32].

Nguyen et al. focused on leptomeningeal metastasis and reported on the findings of different ongoing studies evaluating ICIs in this context. The researchers provided an interim analysis of the Brastianos et al. study, with 44% of patients alive at 3 months after pembrolizumab treatment for solid tumor leptomeningeal metastasis [33, 34]. The use of ICIs in this setting was also the topic of a review by Kondoh et al. [35].

For NSCLC, Gio et al. reported the efficacy of nivolumab in treating leptomeningeal metastasis and did not report any neurological irAEs [36]. Hendricks et al. reported an analysis of 19 patients with leptomeningeal metastases from NSCLC treated with ICIs. No safety data were reported, but the median overall survival was 3.7 months [37]. Nakashima et al. also reported the case of a 66-year-old woman with meningeal carcinomatosis from NSCLC treated with ICIs in combination with whole brain radiation. She achieved more than 23 months of survival without disease progression. This case introduced the idea of including radiotherapy in the treatment regimen. A higher irAE incidence with radiotherapy has not been reported [38–42].

These articles underline the importance of ICIs for the treatment of metastatic CNS tumors and confirm that there is no obvious increase in the incidence of irAEs after such treatment.

## Discussion

### Clinical signs and diagnosis

Neurological irAEs can present as various symptoms [43, 44]. In particular, CNS symptoms are easily underestimated because they manifest at a lower intensity than related symptoms. Usually, neurological irAEs are described in three categories: encephalitis, aseptic meningitis and multiple sclerosis. Nonspecific isolated symptoms, such as headaches, are the most frequently reported symptoms (55%) and are usually low intensity [45].

Other than isolated symptoms, encephalitis and encephalopathy are the most frequently reported irAEs. Regardless, they occur in less than 1% of patients treated with ICIs [6]. Medical practitioners must be aware of these complications, especially due to the broad range of symptoms that can occur. Indeed, unexplained paucisymptomatic headache or focal weakness can be manifestations of grade 1 CNS irAEs [10]. Larkin et al. reported 6 cases of encephalitis, and most patients presented with mental disturbance, seizure and fatigue. Five of the six patients required prolonged hospitalization, and one of them died from complications [10]. Encephalitis leads to increased major morbidity and mortality, especially in cases of limbic encephalitis and cerebral inflammation, even with the administration high-dose steroids [46, 47]. Some pharmacovigilance databases have revealed a fatality rate of 19% when the brainstem is involved [48, 49]. The distinction between neurological irAEs and CNS infection can be challenging, particularly due to the lack of specific positive criteria and the presentation of flu-like symptoms in some cases of irAEs [50]. Infection can also probably induce neurological irAEs, as reported in some cases after herpes simplex infection or Epstein–Barr infection [49, 51]. Ultimately, the diagnosis should be based on a systematic approach with MRI, lumbar puncture, electroencephalography (EEG) if clinically indicated, and screening for typical autoimmune antibodies and/or infectious causes is necessary (Herpesviridae, enterovirus, varicella, and/or bacterial culture) [53, 54]. Nonspecific inflammatory signs can be revealed on MRI and can be consistent with the presence of lymphocytic or neutrophilic pleocytosis, leading to the overlapping diagnosis of immune-induced meningoencephalitis. Of note, all of these tests can also yield normal results; ultimately, patient history and symptom resolution with corticosteroid therapy are factors indicative of a diagnosis of immune-related encephalitis [8].

The second most common CNS irAE described in the series was aseptic meningitis, which was more common with ICI combinations, especially combinations with ipilimumab. Immune-related aseptic meningitis occurred earlier than other neurological irAEs, with a median duration of two cycles and a delay of 9 days from the last injection of ICI to the manifestation of clinical signs [7, 45, 55, 56]. Immune-related aseptic meningitis occurs in less than 1% of cases and represents 6 to 15% of all neurological irAEs [5, 45, 57]. The clinical presentation varies from headache with photophobia to complete cranial hypertension with seizure. This variability in symptoms can make it difficult to distinguish aseptic meningitis from encephalitis. MRI results are often normal or reveal leptomeningeal inflammation. Lumbar puncture usually shows lymphocytosis with elevated protein, which is defined according to ESMO as a white blood cell count between 5 and 500/µL [7]. The CSF is sterile and negative for cytopathology. There are several overlapping diagnostic algorithms used to facilitate the differential diagnosis of immune-related meningitis [8, 58–60]. When testing for encephalitis, lumbar puncture and MRI with infectious disease screening (in particular, PCR for herpes simplex virus but also typical bacterial screening) are essential [61]. When peripheral symptoms are associated with central clinical signs, screening for thyroid dysfunction and/or vitamin B12/B9 deficiency is recommended [59].

### Prevention of irAEs and survival outcomes

Because ICIs are almost universally accepted, the prevention of side effects is key to improving the benefit-risk ratio [65, 66]. The incidence of irAEs depends on the ICI, and different strategies have been explored to limit irAEs [67]. The Checkmate 511 study evaluated two combinations of nivolumab and ipilimumab, comparing treatment with nivolumab 1 mg/kg and ipilimumab 3 mg/kg and treatment with nivolumab 3 mg/kg and ipilimumab 1 mg/kg [68]. After 3 years, the number of grade 3–5 irAEs was significantly lower in the second group (48.3% versus 33.9%), without any difference in OS or PFS [68]. Only the irAEs that occurred in at least 10% of their population were actually reported, so specific data on meningitis are not available.

The prognostic value of irAEs has also been evaluated. Patients who developed side effects seemed to have better survival outcomes than those without any adverse effects [69]. Indini et al. showed improvements in both PFS and OS among patients with MM [9]. Shah et al. analyzed survival data from a cohort of patients who were readministered ICIs after irAEs occurred, and they reported the worst OS and PFS outcomes for patients with a shorter time to the development of initial or post-reintroduction irAEs. On the other hand, patients had a lower risk of disease progression if they completed more than 10 weeks of treatment after the resumption of ICIs.

### Reintroduction of ICIs

The reintroduction of ICIs after the resolution of irAEs is still controversial. The National Comprehensive Cancer Network (NCCN), ESMO and the American Society of Clinical Oncology (ASCO) propose reintroducing ICIs only in cases of grade 1 or 2 irAEs [8, 70, 72]. Indeed, some reports have shown that half of the patients with severe irAEs will develop the same or distinct irAEs after the reintroduction of ICIs [74]. However, patients experiencing irAEs could have better OS and PFS outcomes after reintroduction than those who change treatment regimens [75]. A better understanding of the mechanisms of each irAE is clearly required [76–78].

The management and follow-up of patients with irAEs should be specific to the system affected. Indeed, patients with immune-related hepatitis as an irAE seem to be amenable to the reintroduction of ICIs, with more than 60% of patients avoiding recurrence of grade 2 or greater hepatitis in the study of Allouchery et al. [79]. In contrast, Simonaggio et al. reported that 55% of their patients experienced irAEs after reintroduction. In these patients, colic, pulmonary, joint and hematological toxicities were most likely to occur [74]. Dolladille et al. also explored the characteristics of irAEs after the reintroduction of ICIs, and the results showed that colitis and pneumonitis had higher recurrence rates than rarer irAEs, such as endocrine irAEs [80]. Although there are more than 400 reported irAEs, the rarity of CNS events complicates their analysis. The severity of irAEs, systems affected by irAEs, alternative therapeutic strategies and patient preference must be considered before the resumption of ICIs.

Regarding immune-related meningitis, case reports tend to show that reintroduction of ICIs is possible and can achieve good outcomes. Different strategies can be used, particularly for dual therapy. The reintroduction of ipilimumab has remained controversial because anti-CTLA4 agents are associated with a higher rate of meningitis and irAEs [7, 67]. Albandar et al. also studied survival outcomes after the reintroduction of ICIs, and they reported a median OS of 38.6 months among patients in whom treatment was reinitiated after interruption versus 24.9 months among patients in whom treatment was discontinued. However, this difference was not significantly different [82]. Only a few studies exploring the possibility of ICI reintroduction have been reported, so further studies are needed to help better understand and manage these meningeal irAEs.

## Conclusion

With the emergence of ICIs, AEs have become a new challenge for specialists. In this review, we attempted to describe the variety of clinical signs and consequences of neurological irAEs. Due to their rarity, particularly meningitis, the guidelines recommend systematic biological and clinical examinations to avoid misdiagnosis. Steroids remain the principal treatment for neurological irAEs and successfully resolve the majority of cases. However, whether ICIs should be reintroduced remains to be determined. The answer seems to depend on the system involved, kinetics of improvement and clinical severity, but good outcomes have been achieved after reintroduction in some patients with immune-related meningitis. The collection of additional data in the near future will help to personalize the management strategy and follow-up schedule for patients with such irAEs. In conclusion, our review provides a comprehensive summary of the real-world knowledge on immune-related aseptic meningitis, which we hope will provide guidance for physicians who manage these patients.

## Decalartions

## Conflict of interest

None declared.

## Data Availability

All data analyzed during this study are included in this published article and its supplementary information files.

## Abbreviations

ASCO: American Society of Clinical Oncology
CNS: Central nervous system
CSF: Cerebrospinal fluid
CTLA-4: Cytotoxic T-lymphocyte-associated protein 4
EEG: Electroencephalography
ESMO: European Society of Medical Oncology
FDA: Food and Drug Association
ICI: Immune checkpoint inhibitor
IrAEs: Immune-related adverse events
MM: Metastatic melanoma
MRI: Magnetic resonance imaging
NCCN: National Comprehensive Cancer Network
NMDA: N-methyl-D-aspartate
NSCLC: Non-small-cell lung cancer
OS: Overall survival
PD(L)1: Programmed death (ligand) 1
PET-CT: Positron emission tomography-computed tomography
PFS: Progression-free survival
